# Spine impairment in mice high-expressing neuregulin 1 due to LIMK1 activation

**DOI:** 10.1038/s41419-021-03687-8

**Published:** 2021-04-14

**Authors:** Peng Chen, Hongyang Jing, Mingtao Xiong, Qian Zhang, Dong Lin, Dongyan Ren, Shunqi Wang, Dongmin Yin, Yongjun Chen, Tian Zhou, Baoming Li, Erkang Fei, Bing-Xing Pan

**Affiliations:** 1 School of Life Sciences, Nanchang University, Nanchang, 330031 China; 2 Institute of Life Science, Nanchang University, Nanchang, 330031 China; 3 School of Basic Medical Sciences, Nanchang University, Nanchang, 330031 China; 4 Key Laboratory of Brain Functional Genomics, Ministry of Education and Shanghai, School of Life Science, East China Normal University, Shanghai, 200062 China; 5 South China Research Center for Acupuncture and Moxibustion, Medical College of Acu-Moxi and Rehabilitation, Guangzhou University of Chinese Medicine, Guangzhou, 510006 China

**Keywords:** Molecular neuroscience, Schizophrenia

## Abstract

The genes encoding for neuregulin1 (NRG1), a growth factor, and its receptor ErbB4 are both risk factors of major depression disorder and schizophrenia (SZ). They have been implicated in neural development and synaptic plasticity. However, exactly how NRG1 variations lead to SZ remains unclear. Indeed, NRG1 levels are increased in postmortem brain tissues of patients with brain disorders. Here, we studied the effects of high-level NRG1 on dendritic spine development and function. We showed that spine density in the prefrontal cortex and hippocampus was reduced in mice (cto*Nrg1*) that overexpressed NRG1 in neurons. The frequency of miniature excitatory postsynaptic currents (mEPSCs) was reduced in both brain regions of cto*Nrg1* mice. High expression of NRG1 activated LIMK1 and increased cofilin phosphorylation in postsynaptic densities. Spine reduction was attenuated by inhibiting LIMK1 or blocking the NRG1–LIMK1 interaction, or by restoring NRG1 protein level. These results indicate that a normal NRG1 protein level is necessary for spine homeostasis and suggest a pathophysiological mechanism of abnormal spines in relevant brain disorders.

## Introduction

Neuregulin1 (NRG1) is a large family of neurotrophic factors produced by mRNA splicing of a single gene. With an EGF-like domain, it binds to and activates ErbB receptors such as ErbB4, to initiate downstream signaling pathways^1^. NRG1 is produced in excitatory neurons, GABAergic interneurons, and astrocytes in the brain^2–6^. During development, ErbB4 is expressed in interneuron precursor cells and NRG1/ErbB4 signaling plays a role in assembling the GABAergic circuitry, including interneuron migration and differentiation such as axon development and the formation of excitatory synapses onto interneurons and inhibitory synapses onto pyramidal neurons^6–10^. In adult animals, ErbB4 is almost exclusively in GAD+ (glutamate decarboxylase positive) interneurons in the cerebral cortex, hippocampus (HPF), and amygdala and has been shown critical to GABA (γ-aminobutyric acid) release and excitation–inhibition (E–I) balance^11–13^. Besides, ErbB2 and ErbB4 have been implicated in forming excitatory synapses onto pyramidal neurons^14^. Erbb4 in the interneurons is also involved in GABAergic synapses formation and maintenance^7,9^. Interestingly, the SNP (single nucleotide polymorphism) rs7598440 of ErbB4 has been shown to predict GABA levels in the cortex and cerebrospinal fluid (CSF) in healthy subjects^15,16^, suggesting ErbB4 could impact GABA levels in human subjects, in agreement with roles of ErbB4 in GABA circuit development and function from mouse studies.

Both *NRG1* and *ErbB4* are risk genes for brain disorders including major depressive disorder (MDD) and schizophrenia (SZ). A recent GWAS study of 246,363 patients with depression^17,18^ and MAGMA (Multimarker Analysis of GenoMic Annotation) analysis of the aggregated genetic effects identified *NRG1* and *ErbB4* as putative genes associated with depression. On the other hand, earlier family trio studies, case-controlled association and meta-analysis suggested *NRG1* and *ErbB4* as candidate genes for SZ^19–25^. Although SNPs of neither *NRG1* nor *ErbB4* reached genome-wide significance in a large-population GWAS^26^, perhaps as a result of allelic heterogeneity at their loci, existence of haplotypes and/or population stratification. Nevertheless, most SNPs of *NRG1* and *ErbB4* are intronic and thus may alter gene expression. In agreement, both higher and lower levels of NRG1 and ErbB4 were reported in brain samples or peripheral blood of SZ patients^27–32^, or in neurons derived from SZ patients^33^. NRG1-induced phosphorylation of ErbB4 was increased in the postmortem cortex of SZ patients^34^. NRG1 was increased in the peripheral blood of patients with MDD although NRG1 levels were found to be normal or reduced in patients with depression, compared with healthy subjects^35,36^. In a rat model of depression, NRG1 was increased in the prefrontal cortex (PFC) and HPF^37^. In agreement, mutating NRG1 or altering its levels in mice causes hyperactive locomotion and impairs prepulse inhibition, working memory and conditional fear memory^14,21,38–42^. Mice with increased levels of NRG1, which mimic high levels in patients, exhibited impaired PPI, reduced social interaction, and cognitive deficits^10,38,39,41^. One pathological mechanism of increased NRG1 levels is thought to impair glutamate release from pyramidal neurons. However, the impact of NRG1 high-levels on the postsynaptic component remains unknown.

Here, we examined the effects of high-levels of NRG1 on dendritic spines. In cultured neurons, overexpressing NRG1 impaired spine development and maturation. In agreement, cto*Nrg1* mice, which mimic high-levels of NRG1 in excitatory neurons of forebrain in schizophrenic patients, exhibited reduced spine density. Further molecular studies suggest that high-levels of NRG1 impair dendritic spines via LIMK1 activation. Our results indicate a role of NRG1 in spine homeostasis and reveal a potential mechanism of spinopathy in related disorders.

## Results

### Reduced spine density in neurons expressing high levels of NRG1

**Fig. 1:**
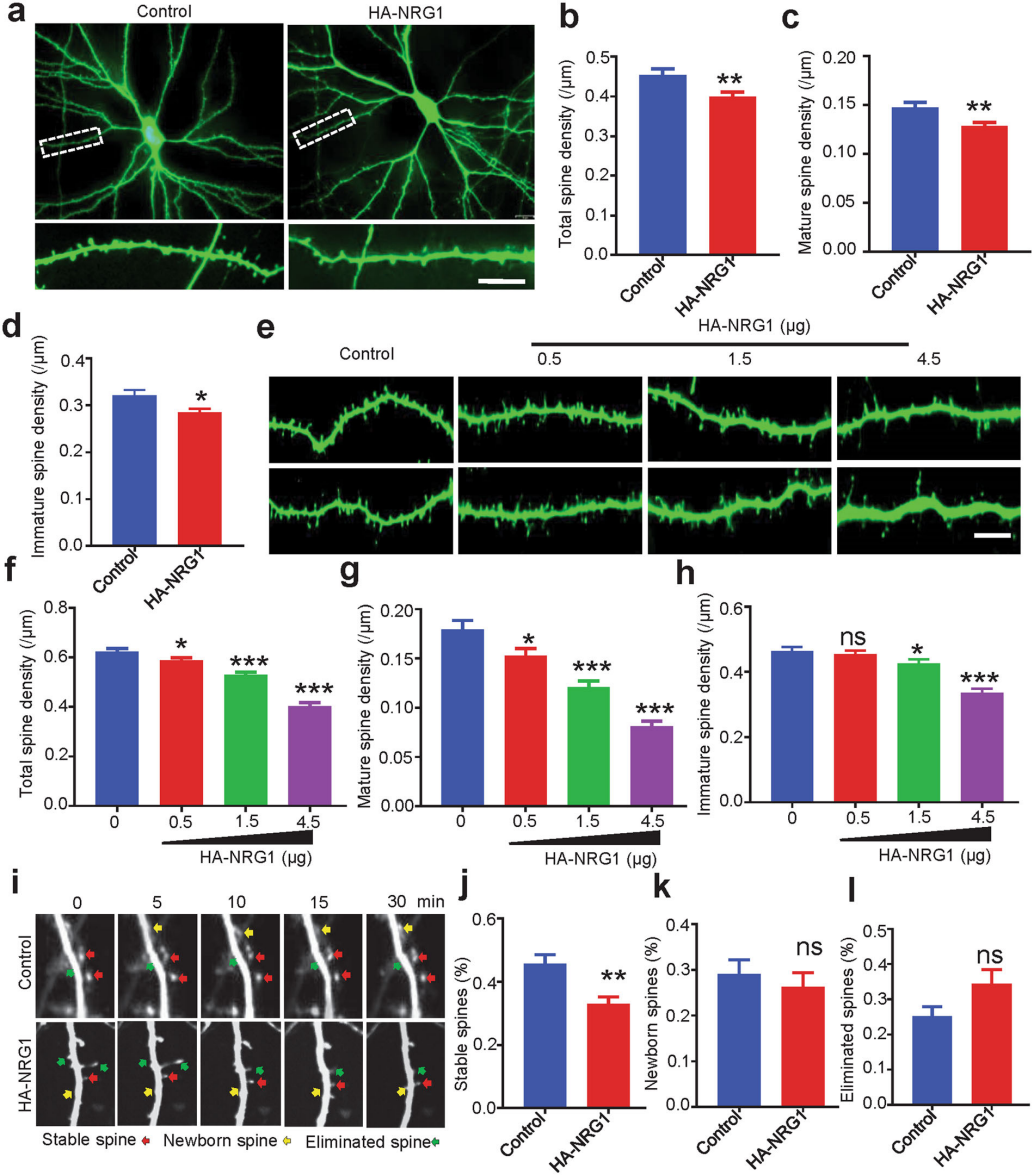
Reduced dendritic spine density in high-expressing NRG1 neurons. **a** Representative images of neuronal morphology and spine density in hippocampal pyramidal neurons. Neurons were isolated at embryonic 18 (E18) rat to culture for 9 days and transfected with 1.5 µg control (empty HA vector) or HA-NRG1 construct, and fixed for staining at DIV17. Scale bar, 10 μm. Statistical analysis of data in **a** for total (**b**), mature (**c**) and immature (**d**) spine density. *N* = 32 neurons for control, *N* = 45 neurons for HA-NRG1 (*p* = 0.0066 for total spine density; *p* = 0.0048 for mature spine density; *p* = 0.0109 for immature spine density). *p < 0.05, and **p < 0.01; Student’s *t*-test. **e** Representative images of spine density in hippocampal neurons transfected with HA-NRG1 in gradient. Scale bar, 10 μm. **f**–**h** The statistical results for total (**f**), mature (**g**) and immature (**h**) spine density. *N* = 28 neurons for control, *N* = 32 neurons for 0.5 μg HA-NRG1, *N* = 31 neurons for 1.5 μg, *N* = 34 neurons for 4.5 μg (*p* = 0.0169 for 0.5 μg, *p* < 0.001 for 1.5 μg and 4.5 μg for total spines; *p* = 0.0251 for 0.5 μg, *p* < 0.001 for 1.5 and 4.5 μg for mature spines; *p* = 0.6044 for 0.5 μg, *p* = 0.0446 for 1.5 μg and *p* < 0.001 for 4.5 μg for immature spines). Data were shown as mean ± SEM; *p < 0.05, **p < 0.01, and ***p < 0.001, one-way ANOVA. **i** Representative images of time-lapse imaging from hippocampal neurons transfected with HA-NRG1 or control taken at five adjacent time points during the 30-min live-imaging period. Cultured neurons were transfected with indicated constructs at DIV9 and imaged every minute for 30-min at DIV17. *N* = 10 neurons for control, *N* = 11 neurons for HA-NRG1. **j**–**l** Quantitative analysis for percentages of stable (red arrow), newborn (yellow arrow) and eliminated (green arrow) spines. *p* = 0.003 for stable spines, *p* = 0.571 for newborn spines, and *p* = 0.07 for eliminated spines. Data were shown as mean ± SEM; **p < 0.01; ns, *p* > 0.05; Student’s *t*-test.

To investigate how the pathological condition of high-levels of NRG1 impact dendritic spines. We transfected HA-tagged, full-length NRG1 and/or GFP into cultured hippocampal neurons at 9 days in vitro (DIV) by calcium phosphate precipitation. At DIV 17–20, neurons were fixed and stained with anti-GFP antibody. The expression of NRG1 increased as we transfected in gradient (Fig. S1). Noticeably, neurons transfected with NRG1 (1.5 µg) displayed reduced total spine density, compared with neurons transfected with empty vector (control) (Fig. 1a, b). The density of mature or mushroom-like (width of spine head/neck > 1.5) and immature (width of spine head/neck < 1.5) spines were both reduced (Fig. 1c, d). The effects of overexpressed NRG1 on spine density were dose-dependent (Fig. 1e–h). Furtherly, we used time-lapse imaging to examine the effects of high-levels of NRG1 on spine dynamics. The same secondary dendritic branch was imaged every minute for 30 min, and percentages of stable, newborn and eliminated spines were analyzed. As shown in Fig. 1i-l, high expression of NRG1 in neurons decreased stable (Fig. 1j), but did not alter newborn and eliminated spines (Fig. 1k, l) during the imaging period. Together, these results strongly suggested high-levels of NRG1 impair spine maturation.

### Reduced spine density and glutamatergic transmission in cto*Nrg1* mice

**Fig. 2:**
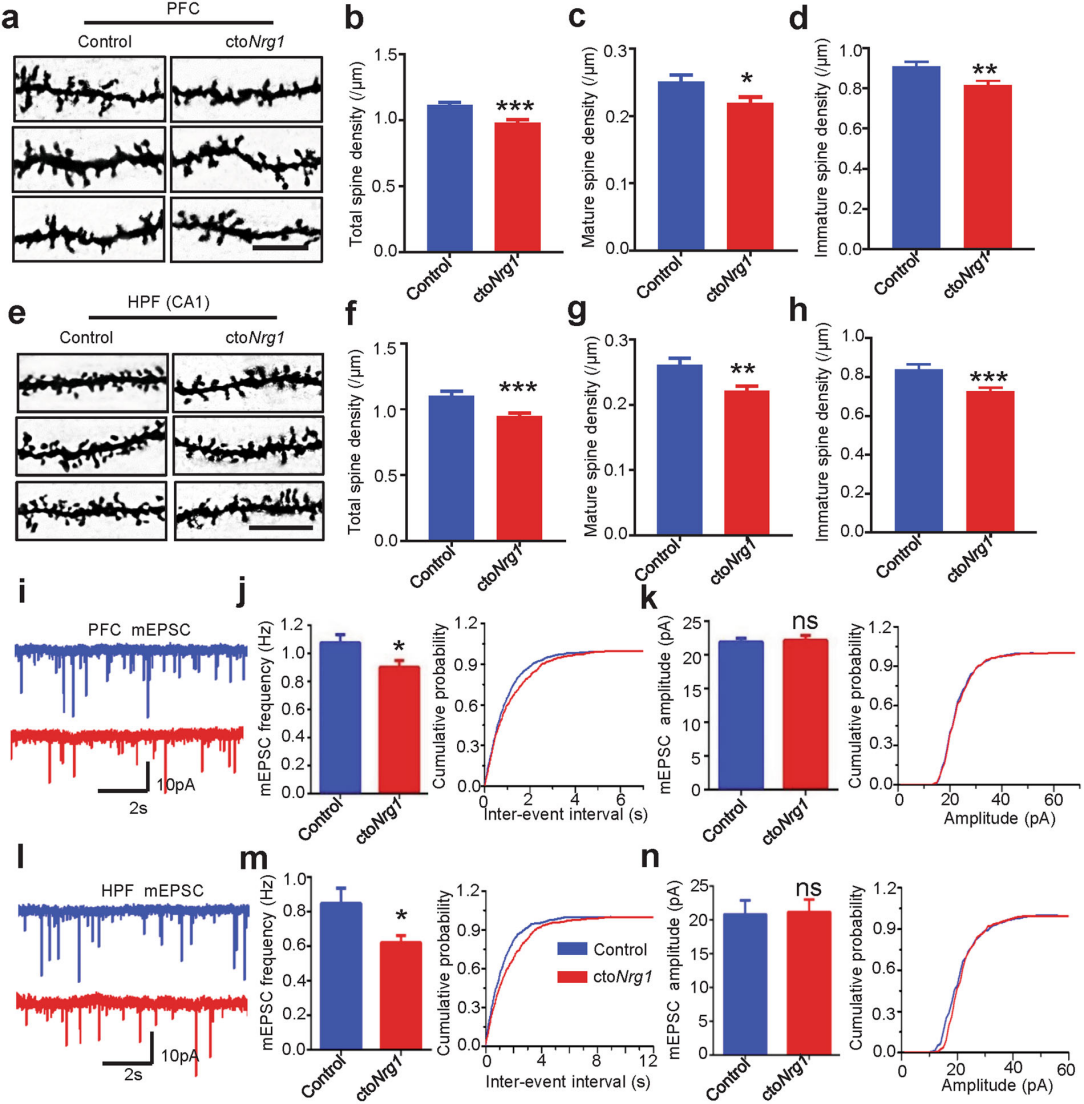
Reduced spine density and glutamatergic transmission in cto*Nrg1* mice. **a**, **e** Representative Golgi staining images of apical dendrites of pyramidal neurons in PFC and HPF. Scale bars,10 μm. **b**–**d** and **f**–**h** Quantitative analysis of total (**b** and **f**), mature (**c** and **g**) and immature (**d** and **h**) spine densities in **a** and **e**. *N* = 5 mice for each genotype (*p* = 0.0017 for total, *p* = 0.0162 for mature and *p* = 0.0028 for immature spine in PFC; *p* = 0.004 for total spine, *p* = 0.0012 for mature and *p* = 0.005 for immature spine in HPF). Data were shown as mean ± SEM; *p < 0.05, **p < 0.01, and ***p < 0.001; Student’s *t*-test. **i**, **l** Representative traces of mEPSCs from pyramidal neurons of PFC prelimbic (PrL) and HPF CA1. Scale bars, 10 pA, 2 s. **j**, **k** and **m**, **n** Histogram summary and cumulative probability plots of mEPSC interevent intervals (**j** and **m**) and amplitude (**k** and **n**) in **i** and **l**. *N* = 13 neurons from three control mice, *N* = 12 neurons from 3 cto*Nrg1* mice in PrL region (*p* = 0.0316 for frequency, *p* = 0.7892 for amplitude); *N* = 14 neurons from three control mice, *N* = 15 neurons from 3 cto*Nrg1* mice in CA1 region (*p* = 0.0197 for frequency, *p* = 0.6902 for amplitude). Data were shown as mean ± SEM. *p < 0.01; Student’s *t*-test.

To determine whether higher levels of NRG1 damage spines in vivo, we characterized cto*Nrg1* mice, compound mice of CaMK2α-tTA and TRE-*Nrg1* mice^10^. TRE-*Nrg1* mice carry HA-tagged type I NRG1β cDNA under the control of the tetracycline-responsive promoter element (TRE) tetO whereas CaMK2α-tTA mice express tTA (tetracycline transactivator) under the control of the CaMK2α promoter (Fig. S2a)^43^. As shown in Fig. S2b, c, different amounts of whole brain lysates (in µg of protein) from cto*Nrg1* and control mice were subjected to western blotting (WB) with anti-NRG1 antibody and NRG1 levels were increased in cto*Nrg1* mice. Furthermore, cto*Nrg1* mice expressed higher levels of NRG1 in pyramidal neurons of the HPF, striatum (STR), PFC, and olfactory bulb (OB), but not thalamus (TH) or cerebellum (CB) (Fig. S2d, e). Overexpression of NRG1 in the HPF, STR, PFC and OB of cto*Nrg1* mice was confirmed by WB with anti-HA antibody (Fig. S2d, e). The level of increase was 30–70% in forebrain regions of cto*Nrg1* mice (Fig. S2d, e), consistent with a previous report^10^. NRG1 overexpression seemed to have little effect on overall brain structure or weight (Fig. S2f–g). Remarkably, total spine densities in the PFC (Fig. 2a, b) and hippocampal CA1 (Fig. 2e, f) were reduced. The mature (Fig. 2c, g) and immature (Fig. 2d, h) spine densities were also decreased. On the other hand, the dendritic length, branches and complexity of pyramidal neurons in PFC (Fig. S3a–d) and CA1 (Fig. S3e–h) were similar between control and cto*Nrg1* mice. These results indicate that NRG1 overexpression impairs spine maturation in neurons of PFC and HPF. In support of this notion was the reduced frequency of miniature excitatory postsynaptic currents (mEPSCs) in both PFC (Fig. 2i–k) and HPF (Fig. 2l–n).

### Activation of LIMK1 by NRG1 overexpression

**Fig. 3:**
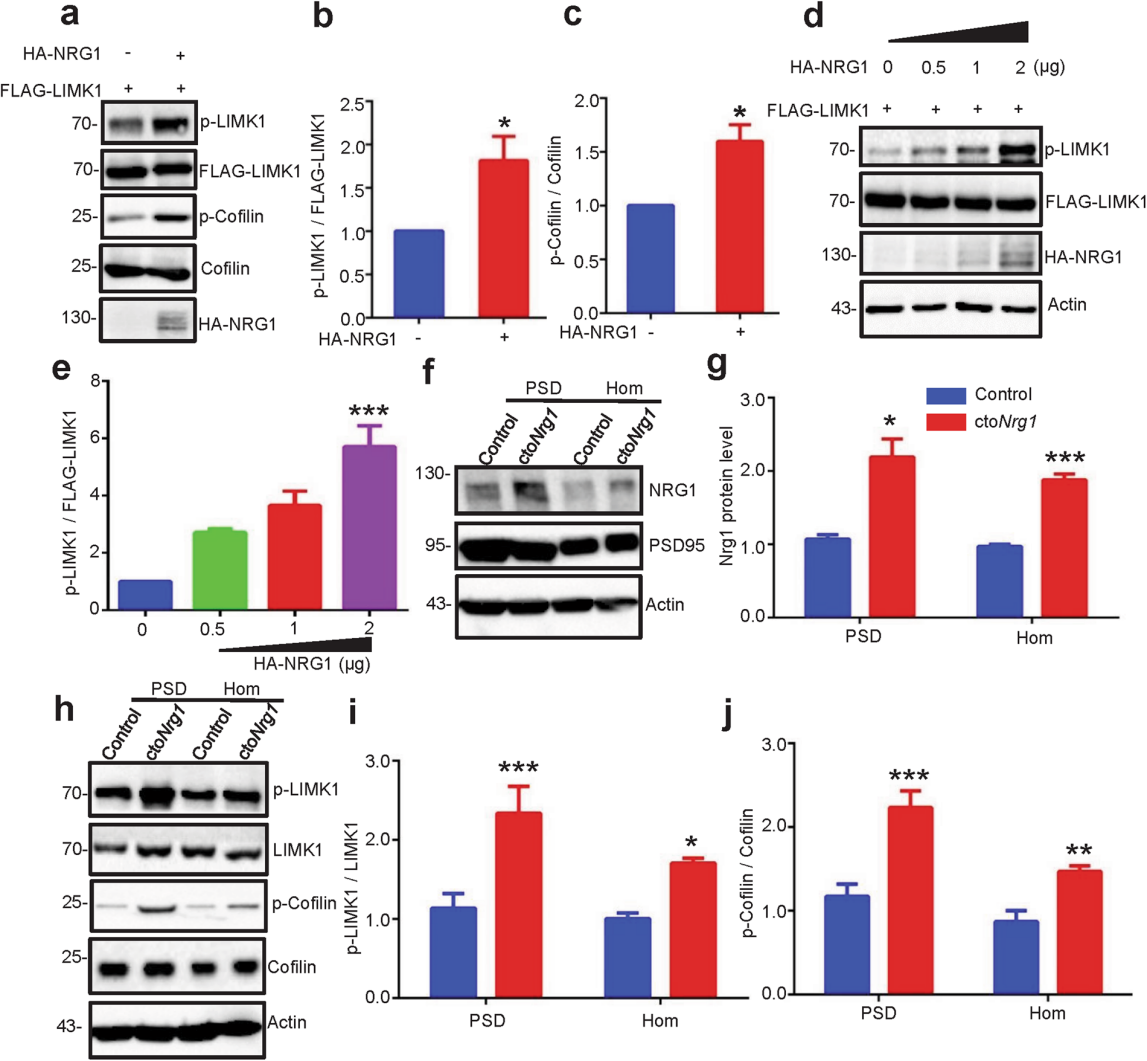
Activation of LIMK1 by NRG1 overexpression. **a**–**c** NRG1 overexpression increased phosphorylations of LIMK1 and its downstream Cofilin in HEK293 cells. HEK293 cells were co-transfected with FLAG-LIMK1 and 1.5 µg HA-NRG1 or HA empty vector and subjected to WB with indicated antibodies (**a**). The relative intensities of phosphorylated LIMK1 (p-LIMK1, Thr505) to FLAG-LIMK1 (**b**) and of phosphorylated Cofilin (p-Cofilin, Ser3) to Cofilin (**c**) from three independent experiments were quantified (*p* = 0.0432 for p-LIMK1; *p* = 0.0185 for p-Cofilin). Data were shown as mean ± SEM. *p < 0.05, Student’s *t*-test. **d**, **e** NRG1 overexpression increased LIMK1 phosphorylation in a dose-dependent manner. FLAG-LIMK1 were co-transfected with different amounts of HA-NRG1 in gradient into HEK293 cells for WB with indicated antibodies. Actin served as a loading control (**d**). Quantitative analysis of relative p-LIMK1 levels in **d** (*p* < 0.001 for 0.5, 1, 2 µg in p-LIMK1) (**e**). Data were from three independent experiments and shown as mean ± SEM. ***p < 0.001, one-way ANOVA. **f**, **g** NRG1 level was increased in the PSDs of cto*Nrg1* mice. Aliquots of whole brain homogenates (Hom. Whole-cell lysates) and PSD fractions from cto *Nrg1* and control mice were probed for NRG1, PSD95 (a PSD marker) and actin (**f**). Quantitative analysis of NRG1 levels in **f** (**g**). **h**–**j** Phosphorylations of LIMK1 and Cofilin were increased in PSD of cto*Nrg1* mice. Representative images of WB with indicated antibodies (**h**). Quantitative analysis of relative p-LIMK1 (**i**) and p-Cofilin (**j**) levels in **h**. *N* = 9 mice for each genotype (*p* = 0.004 in Hom, *p* = 0.0111 in PSD for NRG1 level in **g**; *p* = 0.0176 in Hom, *p* = 0.004 in PSD for p-LIMK1 in **i**; *p* = 0.0088 in Hom, *p* = 0.0005 in PSD for p-Cofilin in **j**). Data were shown as mean ± SEM. *p < 0.05, **p < 0.01, and ***p < 0.001, Student’s *t*-test.

LIMK1 is a serine/threonine kinase that has been implicated in spine development and stability^44,45^. It phosphorylates and thus inactivates Cofilin, an actin depolymerization factor that promotes the turnover and severing of actin filaments^46,47^. The intracellular domain (ICD) of NRG1 interacts with LIMK1^48^. In light of spine deficiency in cto*Nrg1* mice (Fig. 2a–h), we determined whether LIMK1 could be activated by NRG1 overexpression in vitro. HEK293 cells were transfected with FLAG-tagged LIMK1 with or without HA-tagged NRG1 (1.5 µg). As shown in Fig. 3a–c, NRG1 co-expression increased phosphorylated LIMK1 (p-LIMK1, Thr505) and Cofilin (p-Cofilin, Ser3). This effect was dose-dependent (Fig. 3d, e). These results suggest that NRG1 overexpression could activate LIMK1 and inactivate Cofilin. To confirm this effect in vivo, p-LIMK1 and p-Cofilin were detected in cto*Nrg1* mice. NRG1 protein was detectable in both homogenates (Hom, whole-cell lysates) and postsynaptic density (PSD) fraction of both control and cto*Nrg1* mice. Its level was higher in cto*Nrg1* mice than that of control mice (Fig. 3f, g). Remarkably, p-LIMK1 was increased in PSDs of cto*Nrg1* mice, compared with control mice, suggesting that NRG1 overexpression may lead to higher LIMK1 activity (Fig. 3h, i). Likewise, p-Cofilin was increased in cto*Nrg1* PSDs, compared with controls (Fig. 3h, j). Together, these results suggest that NRG1 overexpression activated LIMK1 and thus inactivated p-cofilin in the PSDs in vivo.

### Inactivation of LIMK1 by blocking NRG1–LIMK1 interaction

**Fig. 4:**
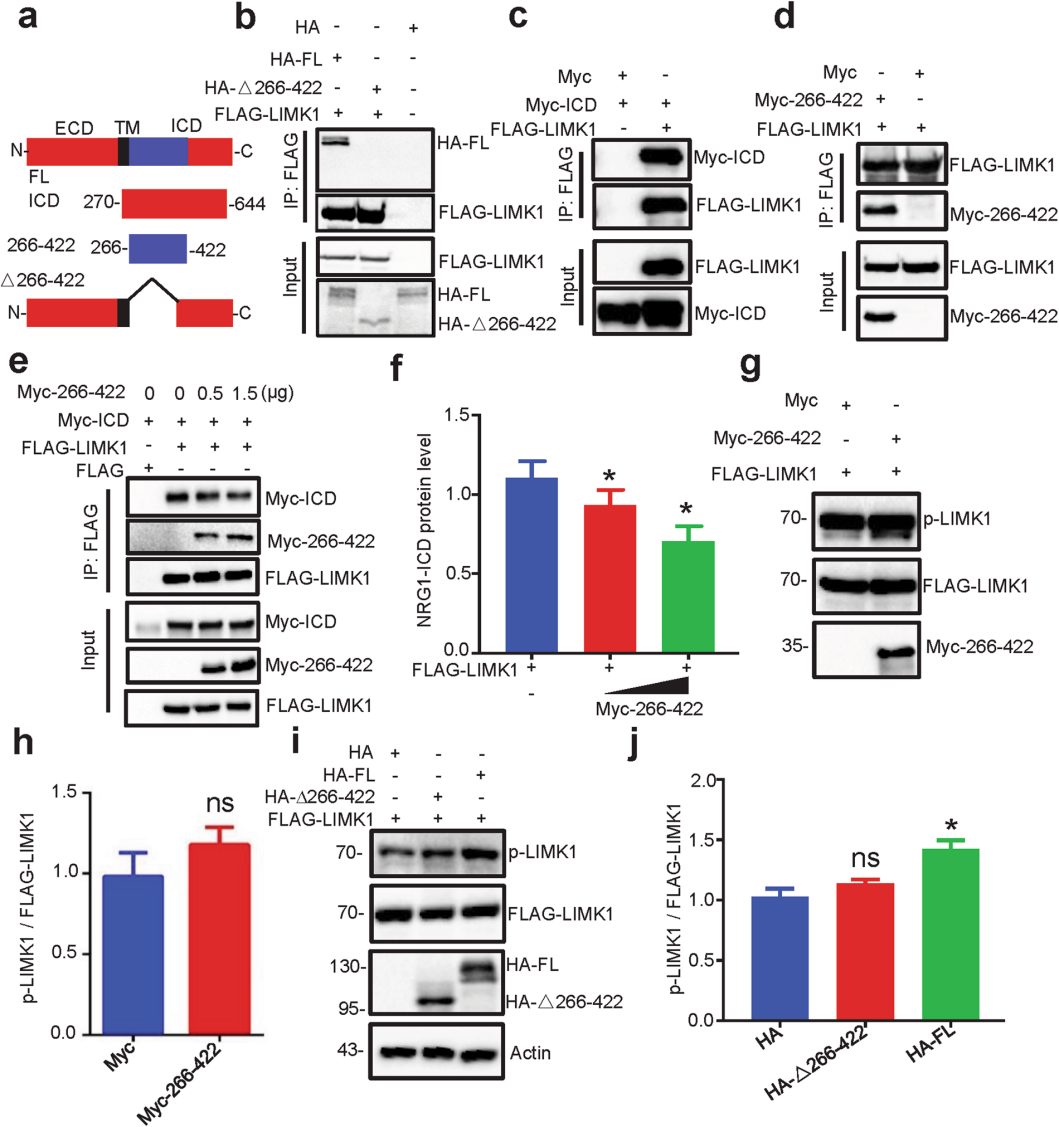
Requirement of NRG1–LIMK1 interaction for LIMK1 activation. **a** Schematic illustration of constructs with different NRG1 domain structures. ECD, extracellular domain; TM, transmembrane domain; ICD, intracellular domain; FL, full length; △266–422, deletion of amino acids 266–422. **b** NRG1 266-422 domain was necessary for NRG1-LIMK1 interaction. HA-FL, HA-△266–422 or HA empty vector were co-transfected with FLAG-LIMK1 into HEK293 cells for immunoprecipitation (IP) with anti-FLAG antibody. **c**, **d** NRG1 266-422 domain was sufficient for NRG1-LIMK1 interaction. Myc-tagged ICD (**c**) or 266-422 (**d**) were co-transfected with FLAG-LIMK1 into HEK293 cells for IP with anti-FLAG antibody. **e**–**h** NRG1 266-422 fragment blocked NRG1–LIMK1 interaction, but not LIMK1 activation. Different amounts of Myc-266-422 were co-transfected with Myc-ICD and FLAG-LIMK1 into HEK293 cells for IP with anti-FLAG antibody (**e**). Quantitative analysis of relative co-IPed NRG1-ICD protein levels in **e** (**f**) (*p* = 0.03405 for 0.5 µg, *p* = 0.01204 for 1.5 µg). Data were from three independent experiments and shown as mean ± SEM. *p < 0.05, one-way ANOVA. HEK293 cells were co-transfected with FLAG-LIMK1 and Myc-266–422 or Myc empty vector for WB with indicated antibodies (**g**). Quantitative analysis of relative p-LIMK1 in **g** (**h**) (*p* = 0.6322 for Myc-266–422). ns: *p* > 0.05, Student’s *t*-test. Data were from three independent experiments and shown as mean ± SEM. **i**, **j** NRG1 without 266-422 domain could not activate LIMK1. HA-FL, HA-△266–422 or HA empty vector were co-transfected with FLAG-LIMK1 into HEK293 cells for WB with indicated antibodies (**i**). Quantitative analysis of relative p-LIMK1 in **i** (**j**) (*p* = 0.2095 for HA-△266–422, *p* = 0.0162 for HA-FL). Data were from three independent experiments and shown as mean ± SEM. ns: *p* > 0.05, *p < 0.05, one-way ANOVA.

The hypothesis that NRG1 interacting with LIMK1 increases its activity predicts that LIMK1 is less active when the NRG1–LIMK1 interaction is blocked. To test this, HEK293 cells were co-transfected with FLAG-tagged LIMK1 and HA-tagged NRG1 derivative constructs (HA-FL, HA-△266-422) (Fig. 4a). As shown in Fig. 4b, HA-FL, but not HA-△266-422, was detectable in the complex precipitated with anti-FLAG antibody, indicating NRG1-ICD interacts with LIMK1 in a manner dependent on the 266–422 fragment, in agreement with a previous report^48^. In addition, we showed that the Myc-tagged NRG1-ICD (Myc-ICD) and 266–422 fragment (Myc-266–422) could be precipitated with anti-FLAG antibody, indicating that this domain was sufficient to interact with LIMK1 (Fig. 4c, d). Having identified the domain required and sufficient to interact with LIMK1, we determined whether this domain was able to inhibit the interaction between NRG1 and LIMK1. HEK293 cells were transfected with increasing concentrations of Myc-266–422 together with Myc-ICD and FLAG-LIMK1. As shown in Fig. 4e, f, the amount of Myc-ICD was reduced in the precipitated LIMK1 complex as Myc-266–422 concentrations increased. These results suggest that the 266–422 fragment can inhibit NRG1–LIMK1 interaction. Notice that the 266–422 fragment alone was unable to alter LIMK1 phosphorylation (Fig. 4g, h), suggesting that this fragment blocks the NRG1–LIMK1 interaction without altering LIMK1 phosphorylation by itself. In addition, NRG1 mutant without the 266-422 (HA-△266-422) was unable to activate LIMK1, suggesting that the interaction of NRG1 and LIMK1 was crucial (Fig. 4i, j). Together, these results suggest the LIMK1 activation requires the interaction with NRG1-ICD.

### Reduced spine deficiency by LIMK1 inactivation and by blocking the NRG1–LIMK1 interaction

**Fig. 5:**
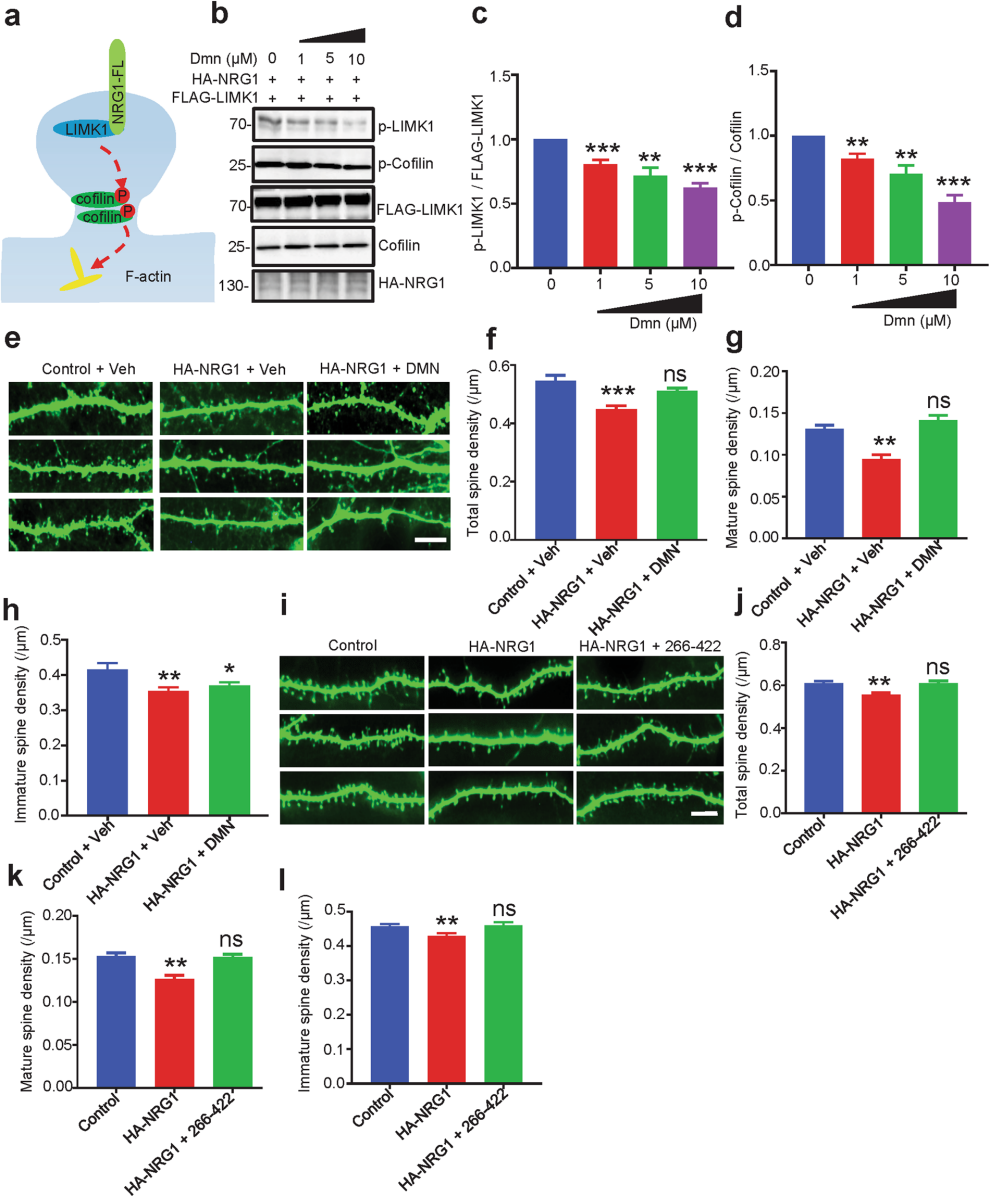
Reduced spine deficiency by LIMK1 inhibition and by blocking NRG1–LIMK1 interaction. **a** A working model shows NRG1 interacted with and activated LIMK1 to affect dendritic spine development in the PSD. **b–d** NRG1-induced LIMK1 activation was inhibited by LIMK1 inhibitor Dmn. HEK293 cells co-transfected with HA-NRG1 and FLAG-LIMK1 were treated with different concentrations of Dmn for 4 h and subjectd to WB with indicate antibodies (**b**). Quantitative analysis of relative p-LIMK1 (**c**) and p-Cofilin (**d**) levels in **b** (*p* = 0.0009 for 1 µM, *p* = 0.004 for 5 µM, *p* < 0.001 for 10 µM in p-LIMK1; *p* = 0.0038 for 1 µM, *p* = 0.0036 for 5 µM, *p* < 0.001 for 10 µM in p-Cofilin). Data were shown as mean ± SEM. *p < 0.05, **p < 0.01, ***p < 0.001, one-way ANOVA. **e**–**h** Spine deficiency in NRG1 high-expressing neurons was rescued by Dmn treatment. Representative images of dendritic spines of cultured neurons. Scale bar, 10 μm (**e**). Primary hippocampal neurons were transfected with HA-NRG1 or control at DIV9 and treated with 10 µM Dmn or its vehicle DMSO for 12 h. Quantitative analysis of total (**f**), mature (**g**), and immature (**h**) spine densities in **e**. *N* = 21 neurons for control + Veh; *N* = 24 neurons for HA-NRG1 + Veh; *N* = 27 neurons for HA-NRG1 + Dmn (*p* < 0.001 for HA-NRG1 + Veh, *p* = 0.0581 for HA-NRG1 + Dmn in total spine; *p* < 0.001 for HA-NRG1 + Veh, *p* = 0.2183 for HA-NRG1 + Dmn in mature spine; *p* = 0.0037 for HA-NRG1 + Veh in immature spine, *p* = 0.0117 for HA-NRG1 + Dmn in immature spine). Data were shown as mean ± SEM. **p < 0.01, ***p < 0.001, one-way ANOVA. **i**–**l** Spine deficiency in NRG1 high-expressing neurons was rescued by NRG1 266-422 fragment. Representative images of dendritic spines of cultured neurons (**i**). Scale bar, 10 μm. Hippocampal neurons (DIV9) were transfected with HA-NRG1 or HA-NRG1 plus 266-422, and fixed at DIV17 for immunostaining. Quantitative analysis of total (**j**), mature (**k**) and immature (**l**) spine densities in **i**. *N* = 27 neurons for control, *N* = 35 for HA-NRG1, *N* = 29 neurons for HA-NRG1 + 266-422 (*p* = 0.006 for HA-NRG1, *p* = 0.9457 for HA-NRG1 + 266–422 in total spine; *p* = 0.0015 for HA-NRG1, *p* = 0.8421 for HA-NRG1 + 266–422 in mature spine; *p* = 0.007 for HA-NRG1, *p* = 0.8374 for HA-NRG1 + 266–22 in immature spine). Data were shown as mean ± SEM. ns, *p* > 0.05, **p < 0.01, and ***p < 0.001, one-way ANOVA.

Our results suggested that high-levels of NRG1 activate LIMK1 activity which was associated with spine density reduction (Fig. 5a). To demonstrate a causal relationship, we determined whether NRG1 overexpression-mediated spine deficits could be attenuated by reducing LIMK1 activity. First, neurons were treated with damnacanthal (Dmn), an anthraquinone derivative that inhibits LIMK1 and Lck, but not CaMK2a, ROK, PKCα, or PAK3^49^. As shown in Fig. 5b–d, Dmn inhibited LIMK1 and Cofilin phosphorylation in a dose-dependent manner. Treatment with 10 µM Dmn, a concentration that effectively inhibits LIMK1, but not Lck^49^, increased the spine densities in hippocampal neurons overexpressing HA-NRG1, compared with neurons treated with vehicle (DMSO) (Fig. 5e–h). These results support the hypothesis that NRG1 overexpression causes spine deficiency by activating LIMK1. Next, we studied the effect of the 266–422 fragment, which could block the NRG1–LIMK1 interaction and thus reduce LIMK1 activity (Fig. 4e–j). Hippocampal neurons transfected with HA-NRG1 exhibited reduced spine densities, compared with control neurons. This inhibitory effect was blocked by co-expressing the 266–422 fragment (Fig. 5i–l). The spine density decreased and with no effect for dendritic length in neurons overexpressing this fragment alone (Fig. S4a–d). A parsimonious explanation of these results is that NRG1 overexpression causes spine deficits by activating LIMK1 and inactivating Cofilin.

### Recovery of spine density in Dox-treated cto*Nrg1* mice

**Fig. 6:**
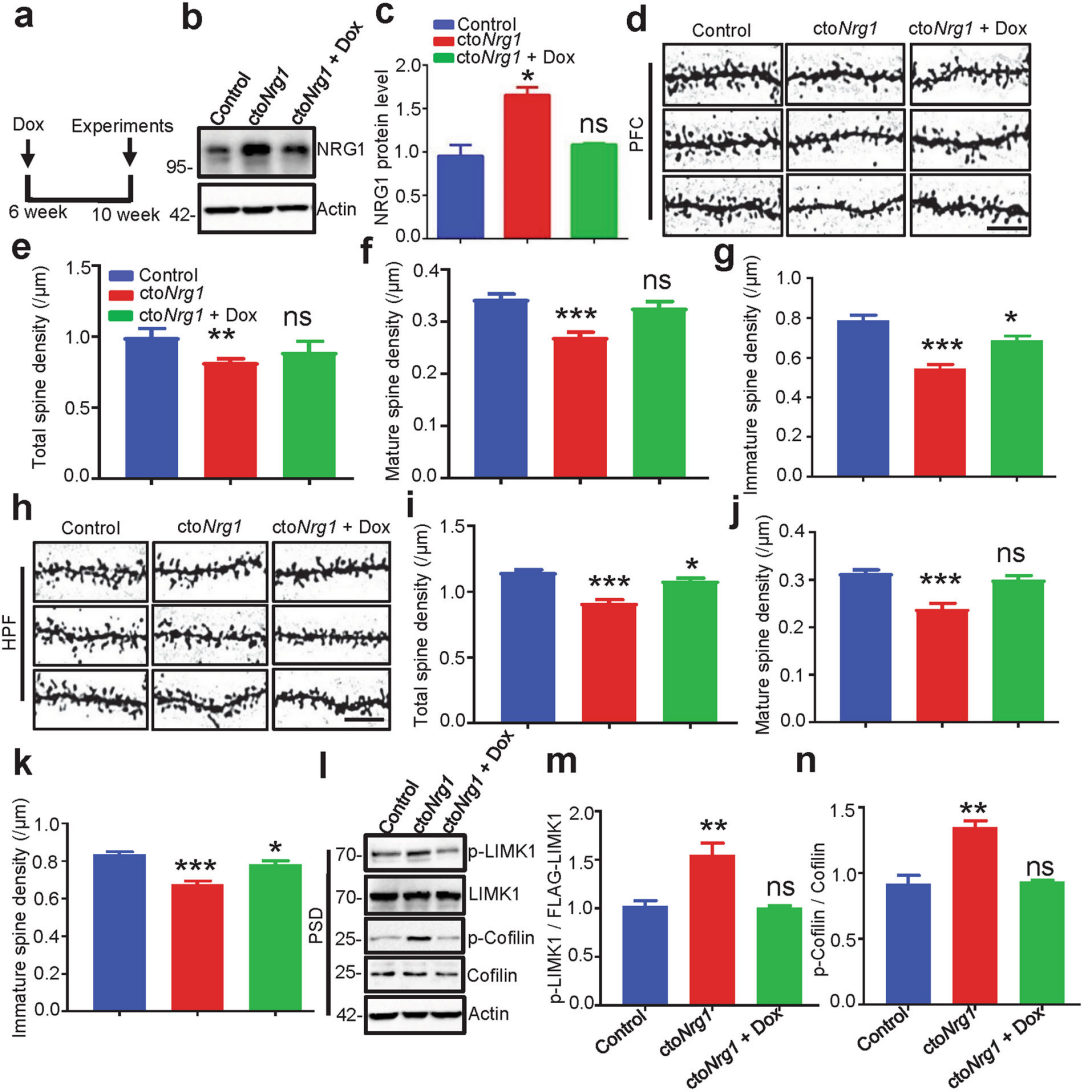
Rescued spine deficiency in cto*Nrg1* mice by restoring NRG1 level. **a** Schematic schedule of Dox treatment. Male 6-week cto*Nrg1* mice were treated with Dox or water for 4 weeks and subjected to WB and Golgi staining. **b**, **c** Increased NRG1 level in the forebrain of cto*Nrg1* mice was restored after Dox treatment. Forebrain lysates from cto*Nrg1*, cto*Nrg1* treated with Dox or control mice were probed with anti-NRG1 antibody. Quantification of NRG1 level (**c**), *N* = 3 mice for each group (*p* = 0.0101 for cto*Nrg1*, *p* = 0.363 for cto*Nrg1* + Dox). Data were shown as mean ± SEM. *p < 0.05, one-way ANOVA. **d**–**k** Reduced spine densities in PFC (**d**–**g**) and HPF (**h**–**k**) of cto*Nrg1* mice were rescued after Dox treatment. Representative Golgi staining images for spine densities in control, cto*Nrg1*, and cto*Nrg1* + dox mice. Scale bar, 10 μm (**d** and **h**). Quantitative analysis of total (**e** and **i**), mature (**f** and **j**) and immature (**g** and **k**) spine densities in **d** and **h**. *N* = 3 mice for each group (In PFC: *p* < 0.001 for cto*Nrg1* and *p* = 0.2265 for cto*Nrg1* + Dox for total spine, *p* < 0.001 for cto*Nrg1* and *p* = 0.5548 for cto*Nrg1* + Dox for mature spine, *p* < 0.0001 for cto*Nrg1* and *p* = 0.013 for cto*Nrg1* + Dox for immature spine; in HPF: *p* < 0.001 for cto*Nrg1* and *p* = 0.0182 for cto*Nrg1* + Dox for total spine, *p* < 0.001 for cto*Nrg1* and *p* = 0.2532 for cto*Nrg1* + Dox for mature spine, *p* < 0.0001 for cto*Nrg1* and *p* = 0.0259 for cto*Nrg1* + Dox for immature spine). Data were shown as mean ± SEM. *p < 0.05, **p < 0.01, and ***p < 0.001, one-way ANOVA. **l**–**n** LIMK1 and Cofilin phosphorylation in PSDs of cto*Nrg1* were restored after Dox treatment. PSD fractions of control, cto*Nrg1*, and cto*Nrg1* + Dox mice were probed with indicated with antibodies (**l**). Quantitative analysis of relative levels of p-LIMK1 and p-Cofilin in **l** (**m**, **n**). *N* = 5 mice for each group (*p* = 0.0087 for cto*Nrg1*, *p* = 0.7758 for cto*Nrg1* + Dox in p-LIMK1; *p* = 0.0019 for cto*Nrg1*, *p* = 08239 for cto*Nrg1* + Dox in p-Cofilin). Data were shown as mean ± SEM. **p < 0.01 and ns, *p* > 0.5. one-way ANOVA.

To determine whether NRG1 overexpression-induced spine deficiency is reversible, cto*Nrg1* mice were treated with Dox (1 mg/kg in drinking water) at the age of 6 weeks for 4 weeks (Fig. 6a). Compared with cto*Nrg1* mice with regular water, NRG1 level was recovered to normal level in the forebrain of Dox-treated cto*Nrg1* mice (Fig. 6b, c). NRG1 levels were similar between TRE-*Nrg1* (control) and Dox-treated cto*Nrg1* mice, suggesting that NRG1 was reduced to a normal level after Dox treatment. Noticeably, the spine density and maturation in Dox-treated cto*Nrg1* mice were increased, compared with those in untreated cto*Nrg1* mice, in both PFC (Fig. 6d–g) and HPF (Fig. 6h–k), indicating that spine deficiency by NRG1 overexpression could be rescued by reducing NRG1 levels. The spine densities of Dox-treated cto*Nrg1* mice remained lower than those in control mice in the HPF, suggesting that the rescue effect was partial. However, in the PFC, there was no difference in spine densities between control and Dox-treated cto*Nrg1* mice, suggesting a complete rescue. In addition, the LIMK1 and its downstream Cofilin phosphorylation were also restored to the normal level compared with control in the PSD area of cto*Nrg1* mice treated with Dox (Fig. 6l, m). Together, these results suggest that high-levels NRG1-mediated spine deficiency could be attenuated by reducing NRG1 levels in young adult mice and suggest that NRG1 is critical in regulating spine density.

## Discussion

Our findings provided a new pathophysiological mechanism of NRG1 for SZ. First, spine density and its maturation were reduced in cultured neurons high-expressing NRG1 (Fig. 1). The spine deficits were also observed in the PFC and HPF of cto*Nrg1* mice overexpressing NRG1 in forebrain excitatory neurons (Fig. 2). Second, high-levels of NRG1 activated LIMK1 and inactivated Cofilin in vitro and in vivo (Fig. 3). Third, either inhibiting LIMK1 activity or blocking NRG1–LIMK1 interaction attenuated NRG1 overexpression-induced spine deficits (Fig. 5). These observations demonstrate that spine development requires proper levels of NRG1 and high-levels of NRG1 impair spine formation and maturation. These results may contribute to our understanding of mechanisms of NRG1 participating in relevant brain disorders.

The cytoskeleton of the dendritic spine is formed by filamentous actin (F-actin), which supports the spine shape and drives the postsynaptic signaling pathway to maintain spine stability and dynamic^50,51^. The small GTPases of Rho family, mostly including RhoA, Rac1, and Cdc42, promote or suppress the actin polymerization by active GTP-bound and inactive GDP-bound change states to regulate spine morphogenesis^52–54^. Downstreams of Rho GTPases include LIMK1, Wiskott–Aldrich syndrome proteins (WASPs), ARP and WASP-family verprolin homologous (WAVEs)^45,55,56^. Rac1 activates the downstream effectors p21-activated kinase (PAK), LIMK1, and F-actin-binding protein Cofilin to regulate actin polymerization and stabilize dendritic spines^57,58^. Spine morphology and development are impaired by LIMK1 deficiency or miR-134-mediated inhibition of LIMK1 translation^45,59^. LIMK1 is a serine/threonine kinase that regulates actin dynamics by phosphorylating its downstream Cofilin^46,47^. NRG1 via its ICD interacts with LIMK1^60^, and high-levels NRG1 recruit LIMK1 into synaptic areas for overactivation to impair synaptic transmission in cto*Nrg1* mice^10^. Phosphorylated LIMK1 and Cofilin were also increased in the PSDs of cto*Nrg1* mice (Fig. 3h–j). We showed that NRG1 overexpression induced spine deficits could be partially restored by inhibiting LIMK1 activation (Fig. 5e–h) or NRG1–LIMK1 interaction (Fig. 5i–l). It is very difficult to discriminate if presynaptic LIMK1/Cofilin signaling was involved in dendritic spine maturation in vivo. Even though some have reported that presynaptic signaling is crucial for synaptogenesis, dendritic spine formation and maintenance are normal in the absence of presynaptic neurotransmitter secretion^61^. Reducing NRG1 protein levels in cto*Nrg1* mice reduced LIMK1and Cofilin phosphorylations and attenuated spine deficits (Fig. 6). However, the rescue experiments by crossing cto*Nrg1* with LIMK1 knockout mice could be performed to validate this mechanism in vivo.

In postmortem schizophrenic patients, spine density is decreased from 23% to 66% compared with normal control in PFC layer 3 pyramidal neurons^62–64^, but not for layers 5 and 6^65^. The spine volume is decreased by 35%, and the total spine number is decreased by 47% in the schizophrenic hippocampal CA3 region^66^. Spine deficit might be a significant hallmark for SZ. Here we also observed spine development deficits in cto*Nrg1* mice, which display SZ-related abnormal behaviors. Interestingly, it has been reported that overexpressing type-III NRG1 under the Thy1.2 promoter in mice causes abnormal spine morphology, but normal spine density^67^. Due to the unstable expression pattern of Thy1.2 promoter^68^, it might not be an ideal model to mimic high expression levels and regions of NRG1 in schizophrenic patients. However, in our cto*Nrg1* mice, NRG1 was overexpressed under CamK2α promoter and in a tTA-induced manner (Fig. S2a). It has been shown that cto*Nrg1* mice increase NRG1 level by 50–100% in the forebrain, similar to that in schizophrenic forebrains. So, the cto*Nrg1* mice are a relatively better model for mimicking high-levels of NRG1 under SZ pathological conditions.

Previous studies suggested that elevated NRG1 levels or signaling are associated with SZ. The NRG1 mRNA and protein levels are increased in the PFC and HPF of schizophrenic patients^27,28,69,70^. Mimicking high levels of NRG1 in mice also results in relevant behavioral deficits^10,38,39,41^. Continuous high expressing NRG1 leads to impaired glutamatergic and GABAergic transmission^10^. Recently, it has been reported that *NRG1* and *ErbB4* are both risk genes for MDD^17,18^. Although NRG1 mRNA level is increased in the peripheral blood of patients with MDD, its level in the brain is still unclear^35,36^. And dysregulation of the NRG1 level has been observed in different rodent depression models. NRG1 protein level is increased in the PFC and HPF in a rat model of chronic unpredictable mild stress (CUMS)^37^. However, in the mouse model of chronic social defeat stress (CSDS), NRG1 protein level was decreased in medial PFC (mPFC) and HPF^71,72^. Moreover, overexpressing NRG1 in mPFC through virus attenuates depressive-like behaviors in CSDS mice, suggesting NRG1 deficiency in mice mPFC played a key role for stress susceptibility^72^. Therefore, NRG1 plays a critical role in depression based on its protein levels. Interestingly, the phosphorylation levels of LIMK1 and Cofilin, but not protein levels, are increased in the mPFC of the CUMS, CSDS and chronic restraint stress (CRS) mouse models^73^. Considering spine synapses density is decreased in the dorsolateral PFC (dlPFC) layer 2/3 of MDD patients^74^, high-levels NRG1 induced LIMK1 activation might also contribute to the spine deficits in MDD. In the future, cto*Nrg1* mice could be exposed to stress and subjected to depressive-like behavioral tests to detect if high-levels NRG1 induce stress susceptibility. Taken together, NRG1 plays a critical role in the central nervous system based on its gene–dosage balance, and abnormal levels or activity of NRG1 could potentially contribute to the pathogenesis of relevant neurological disorders.

## Materials and methods

### Animals

Cto*Nrg1* mice were described as previously^10^. All mice were housed in a constant temperature and humidity chamber at 23 °C, and sufficient food and water were administered daily. No more than five adult mice per cage were subjected to a 12-h light/dark cycle under standard conditions. All the mice were guaranteed to be hygienic. The animal experiments were carried out following the “Guidelines for the Care and Use of Laboratory Animals” promulgated by Nanchang University.

### Cell culture and transfection

Human embryonic kidney (HEK) 293 cells were cultured in Dulbecco’s modified Eagle’s medium (DMEM) (Gibco) supplemented with 10% fetal bovine serum (FBS) (Gibco). Transient transfection was performed using polyethylenimine (PEI) (Sigma, 408727), as described before^75^. Briefly, cells were cultured in 100 mm dishes and at ∼70% confluence were incubated with precipitates formed by 5 μg of plasmid DNA and 280 μL of 0.05% PEI (wt/vol). Cells were harvested 24–48 h post-transfection.

Cultures of primary hippocampal neurons were prepared from embryonic day (E) 18 Sprague-Dawley rats as described previously^8^. Briefly, hippocampi were isolated and kept separate from one another in HBSS on ice. Following digestion in 0.25% trypsin plus 0.1 mg/mL DNase I (one HPF in 1 mL) at 37 °C for 20 min. Dissociated cells were resuspended in plating media (DMEM supplemented with 10% FBS) and plated at a density of 1 × 10^5^ or 2 × 10^5^ per well onto poly-d-lysine-coated 20-mm coverslips (WHB) in 12-well plates (Corning). Cells were incubated for 4 h before replacing with maintenance medium [neurobasal medium (Gibco) supplemented with 2% B-27 supplement (Gibco), 1% GlutaMax (Gibco), and 1% penicillin/streptomycin (Gibco)]. Neurons were maintained at 37 °C in 5% CO_2_, with half of the medium changed every 2–3 days.

For transfection in neurons, calcium phosphate precipitation was performed as described previously^75^. Briefly, the neurons were serum-starved with pre-heated DMEM for 2 h at 37 °C in 10% CO_2_. For each well of 12-well plate, 1–6 μg DNA in 1–6 μL was mixed with 5 μL 2.5 M CaCl_2_ in ddH_2_O (total volume 50 μL), and further mixed with 50 μL of Hepes-buffered saline containing (in millimoles): 274 NaCl, 10 KCl, 1.4 Na_2_HPO_4_, 15 glucose, and 42 Hepes, pH 7.05. Resulting DNA–calcium phosphate precipitates were added into neurons. Morphology was studied 3–7 days later.

### Western blotting

For protein expression detection, tissues were homogenized in PBS plus protease and phosphatase inhibitors. Then the homogenates were lysed in equal volume of 2 × RIPA buffer [0.2% SDS (wt/vol), 1% sodium deoxycholate (wt/vol) and 2% Nonidet P-40 (vol/vol) in PBS] plus protease and phosphatase inhibitors. Lysates were centrifuged at 12,000 × *g* for 20 min at 4 °C to remove debris. The supernatants were subjected to Bradford assay (Pierce) to measure protein concentration and diluted in SDS sample buffer.

Protein samples (10–20 µg) were resolved by SDS–PAGE and transferred to PVDF membrane (Millipore). The membrane was immunoblotted with primary and secondary antibodies, and immunoreactive bands were visualized by enhanced chemiluminescence under gel documentation system (Bio-Rad). Densitometric quantification of protein band intensity was performed by using ImageJ. Antibodies were diluted with primary antibody dilution buffer (TBS + 1%TritonX-100 + 5%BSA) for WB: anti-HA (Biolengend, mouse,1:500, 901513), anti-FLAG (Sigma, mouse, 1:2000, 1804), anti-Myc (SCTB, mouse, 1:1000, sc-40), anti-Cofilin (SCTB, rabbit, 1:500, sc-33779), anti-*p*-Cofilin (SCTB, rabbit, 1:500, sc-21867R), anti-LIMK1 (mouse, BD, 1:1000, 611748), anti-*p*-LIMK1 (rabbit, cell signaling, 1:1000, 3841), anti-PSD95 (mouse, millipore, 1:1000, MABN1194), anti-Neuregulin-1 (rabbit, SCTB, 1:500, sc-393006), anti-β-actin (rabbit, SCTB, 1:2000,sc-130656) and anti-GFP (mouse, SCTB, sc-9996).

### Immunoprecipitation

Immunoprecipitation was performed as described previously^76^. For co-immunoprecipitation (co-IP), transfected HEK293 cells were lysed in IP buffer containing (in millimoles): 20 Tris, pH7.6, 50 NaCl, 1 EDTA, 1 NaF, 0.5% Nonidet P-40 (vol/vol), with protease and phosphatase inhibitors. Samples were centrifuged at 12,000 × *g* for 20 min at 4 °C to remove debris. Lysates (1–2 mg) were incubated with corresponding antibody (1–2 µg) at 4 °C for either 3–4 h or overnight and then incubated with 10–15 µL Protein A/G magnetic agarose beads (Pierce) at 4 °C for 1 h. Samples were washed with IP buffer and resuspended in SDS sample buffer. Then the samples were subjected to WB.

### Time-lapse imaging and analysis of dendritic spines

Live imaging of cultured neurons was performed as described previously with modifications^77^. Cultured rat hippocampal neurons were transfected by Calcium phosphate precipitation at DIV9 and subjected to live imaging at DIV15. Z-stack images of secondary dendrites from transfected neurons were imaged every minute for 30 min, using an Olympus FV1000 confocal microscope with a ×40 (NA 1.35) objective for time-lapse imaging. Images were collapsed into 2D projections and analyzed with ImageJ software. Stable spines were defined as protrusions with stable morphology during the entire imaging session; newborn spines were those emerging protrusions after imaging, regardless of the time they emerged and whether they persisted during the entire imaging session; eliminated spines were present at the beginning of imaging, but disappeared during the imaging session.

### Immunostaining

Immunostaining was performed as described previously with modifcations^75^. Primary cultured neurons were fixed with 4% paraformaldehyde (PFA)/4% sucrose (wt/vol) for 15 min. After washing three times with PBS, neurons were incubated with primary antibody diluted in GDB buffer (30 mM phosphate buffer, pH 7.4, containing 0.2% gelatin, 0.6% Triton X-100, and 0.9 M NaCl) at 4 °C overnight. After washing three times with washing buffer (20 mM phosphate buffer and 0.5 M NaCl), neurons were incubated with the corresponding Alexa Fluor-conjugated secondary antibodies (diluted in GDB buffer) at room temperature for 1 h. The images were obtained by Olympus, FSX100.

### Subcellular fractionation

Mice brain subcellular fractions were performed as described previously with modifications^76^. Adult mice cerebral cortices were homogenized in 10 volumes of HEPES-buffered sucrose (0.32 M sucrose, 4 mM HEPES/NaOH, pH 7.4) with a glass-Teflon homogenizer. The homogenate (Hom) was centrifuged at 1000 × *g* for 10 min to remove the nuclear fraction and unbroken cells. The supernatant (S1) was then centrifuged at 10,000 × *g* for 15 min to yield the crude synaptosomal fraction and the supernatant (S2). This pellet was resuspended in 10 vol of HEPES-buffered sucrose and then centrifuged at 10,000 × *g* for another 15 min. The resulting pellet (P2) was lysed by hypo-osmotic shock in water, rapidly adjusted to 4 mM HEPES, and mixed constantly for 30 min (on ice). The lysate was then centrifuged at 25,000 × *g* for 20 min to yield the supernatant (S3, crude synaptic vesicle fraction) and a pellet (P3, lysed synaptosomal membrane fraction). The pellet was resuspended in HEPES-buffered sucrose, carefully layered on top of a discontinuous gradient containing 0.8–1.0–1.2 M sucrose (top to bottom), and centrifuged at 150,000 × *g* for 2 h. The gradient yields a floating myelin fraction (G1), a light membrane fraction at the 0.8 M/1.0 M sucrose interface (G2), a synaptosomal plasma membrane (SPM) fraction at the 1.0 M/1.2 M sucrose interface (G3), and a mitochondrial fraction as the pellet (G4). Collect the G3 layer and add equal volume HEPES-buffered sucrose then centrifuged at 20,000 × *g* for 15 min to get the SPM. Resuspending the SPM with 1% Triton X-100 in 50 mM HEPES/NaOH (pH 8) on ice for 15 min and then centrifuged at 20,000 × *g* for 15 min to yield the soluble presynaptic membrane protein and the pellet is the PSD (Soluble in 2%SDS PBS buffer at RT).

### Electrophysiological recordings

Electrophysiological recordings were performed as described previously^10^. Briefly, slices were placed in recording chamber that was perfused (3 mL/min) with ACSF containing (126 mM NaCl, 3 mM KCl, 1.25 mM NaH_2_PO_4_, 1.0 mM MgSO_4_, 2.0 mM CaCl_2_, 26 mM NaHCO_3_, and 10 mM Glucose) at 32–34 °C. Whole-cell recording from the PFC and HPF pyramidal neurons was aided with infrared optics using an upright microscope equipped with a 40 × water-immersion lens (Olympus, BX51WI) and infrared-sensitive CCD camera. The pipette (input resistance: 2–4 MΩ) solution contained 135 mM Cs-methanesulfonate, 8 mM NaCl, 10 mM HEPES, 10 mM phosphocreatine, 4 mM ATP-Mg, 0.3 mM GTP-Na, 0.3 mM EGTA, and 5 mM QX314 (Tocris Bioscience, #0190) (pH, 7.3, 295 mOsm). To measure miniature EPSCs (mEPSCs) were blocked with 20 µM bicuculline methiodide (BMI) (Tocris Bioscience, #0130).

### Golgi staining

Golgi staining was prepared as described previously^78^. The Golgi staining regent (FD Rapid GolgiStainTM Kit, cat: PK401). Briefly, the animal brain should be removed from the skull and rinse tissue quickly in double-distilled to remove blood from the surface. The brain transferred into the impregnation solution made by mixing equal volumes of Solutions A and B, and store at room temperature for 2 weeks in the dark. Add at least 5 ml of the impregnation solution for each brain. Replace the impregnation solution after the first 6 h of immersion or the next day. Transfer brain tissue into Solution C and store it at room temperature of dark for at least 72 h. Replace the solution at least once after 24 h of immersion. 80–100 μm section can be best cut on a cryostat at −25 °C to −27 °C. Brain tissue may also be mounted with any type of tissue freezing medium, such as OCT. Each section should then be transferred with a glass specimen retriever into a 50 mL beaker, the beaker outside coated with aluminum foil, which installed Milli-Q water. The beaker should be stirred gently for the first time, then store at room temperature for a few minutes. Place sections in a mixture consisting of 1 part Solution D, 1 part Solution E and 2 parts double-distilled water for 10 min. Rinse sections in double-distilled water 2 times, 4 minutes each. Dehydrate sections in 50%, 75%, and 95% ethanol, 4 min and 5 mL each. Dehydrate sections in absolute ethanol, four times, 4 min and 5 mL each. The images were obtained by Olympus, FSX100.

### Statistics analysis

Statistical analysis was done by the GraphPad Prism version 6.0 (GraphPad Software). All statistical analyses are presented as mean ± SEM and were analyzed by two-tailed Student’s *t* test and one-way ANOVA including Golgi staining, mEPSCs and WB. Values of *p* < 0.05 were considered statistically significant. Statistical significance was set at *p < 0.05, **p < 0.01, and ***p < 0.001.

## Supplementary information

Supplementary Figure Legends

Supplemental Figure 1

Supplemental Figure 2

Supplemental Figure 3

Supplemental Figure 4
