# Supplementary material for: Spine impairment in mice high-expressing neuregulin 1 due to LIMK1 activation
Source: Cell Death Dis. 2021 Apr 14;12(4):403. doi: 10.1038/s41419-021-03687-8 (PMC8047019; doi:10.1038/s41419-021-03687-8)
Supplement: Supplementary file 1 — Supplementary Figure Legends [file 41419_2021_3687_MOESM1_ESM.docx]

**Supplemental Figure legends**

**Figure S1. Gradual increased NRG1 expression in hippocampal neurons transfected with HA-NRG1 in gradient.**

Representative staining images of transfected neurons. Cultured neurons were transfected with empty vector (control) or HA-NRG1 in gradient plus GFP at DIV9 and fixed for staining with indicated antibodies at DIV17. Scale bar, 10 μm.

**Figure S2. Normal cortical and hippocampal structures of cto*Nrg1* mice.**

**a** Breeding diagram of cto*Nrg1* mice. TRE-*Nrg1* mice were crossed with CamK2α-tTA to generate CamK2α-tTa::TRE-*Nrg1* (cto*Nrg1*) mice. **b-c** Increased NRG1 expression in the brain of cto*Nrg1* mice. Different amounts of whole brain lysates (in µg of protein) from Cto*Nrg1* and control mice were subjected to WB with anti-NRG1 antibody. Gray values of NRG1 bands in **b** were quantified (**c**). N = 3 mice for each genotype (p < 0.001). Data were shown as mean ± SEM. ***p < 0.001, two-way ANOVA. **d-e** Elevated NRG1 expression in the forebrain of cto*Nrg1* mice. Lysates of HPF, striatum (STR), PFC, olfactory bulb (OB), thalamus (TH) and cerebellum (CB) from cto*Nrg1* or control mice were probed with anti-NRG1 and anti-HA antibodies (**d-e**). Quantification of NRG1 level in **d** and **e**. N = 4 mice for each genotype (p < 0.001 for STR, p = 0.0029 for HPF, p = 0.0039 for PFC, p = 0.0025 for OB, p = 0.1298 for TH, and p = 0.3074 for CB). Data were shown as mean ± SEM. ns, p > 0.05, *p < 0.05, **p < 0.01, and ***p < 0.001, Student’s t-test. **d-e** Normal brain weight and overall brain structure in cto*Nrg1* mice. Brains from male adult cto*Nrg1* and control mice were collected for weighing (**d**) and Nissl staining (**e**). Representative brain image and quantificational of brain mass for each genotype (**d**). N = 9 mice for each genotype (p = 0.0997). Data were shown as mean ± SEM. ns, p > 0.05, Student’s t-test. Representative Nissl staining images of cortex and HPF of cto*Nrg1* and control mice (**e**).

**Figure S3. Normal dendritic arborization in PFC and HPF of ctoNrg1 mice.**

**a-h** Male adult cto*Nrg1* and control mice brains were collected for Golgi staining. Representative neuronal images of pyramidal neurons in PFC (**a**) and HPF (**e**), Scale bars, 64 μm. Quantitative analysis of dendritic length (**b** and **f**), branches (**c** and **g**) and Sholl analysis (**d** and **h**) in **a** and **e**. N = 3 mice for each genotype (In PFC, p = 0.5611 for length, p = 0.0.0822 for branches; in HPF, p = 0.5634 for length, p = 0.3591 for branches). Data were shown as mean ± SEM. ns, p > 0.05, Student’s t-test.

**Figure S4. Reduced spine density, but normal dendritic arborization in neurons transfected with NRG1 266-422 alone.**

**a-d** Primary hippocampal neurons were transfected with 266-422 or control plus GFP at DIV9 and fixed for observations of dendritic spines (**a-b**) and dendrite arborization (**c-d**). Representative images of spines (**a**) and traced dendrites (**c**). Quantitative analysis of data in **a** and **c** (p = 0.0463 for total spine, p = 0.832 for dendritic length). Data were shown as mean ± SEM. ns, p > 0.05, *p < 0.05, Student’s t-test.
