# Supplementary figures and images for: Spine impairment in mice high-expressing neuregulin 1 due to LIMK1 activation
Source: Cell Death Dis. 2021 Apr 14;12(4):403. doi: 10.1038/s41419-021-03687-8 (PMC8047019; doi:10.1038/s41419-021-03687-8)

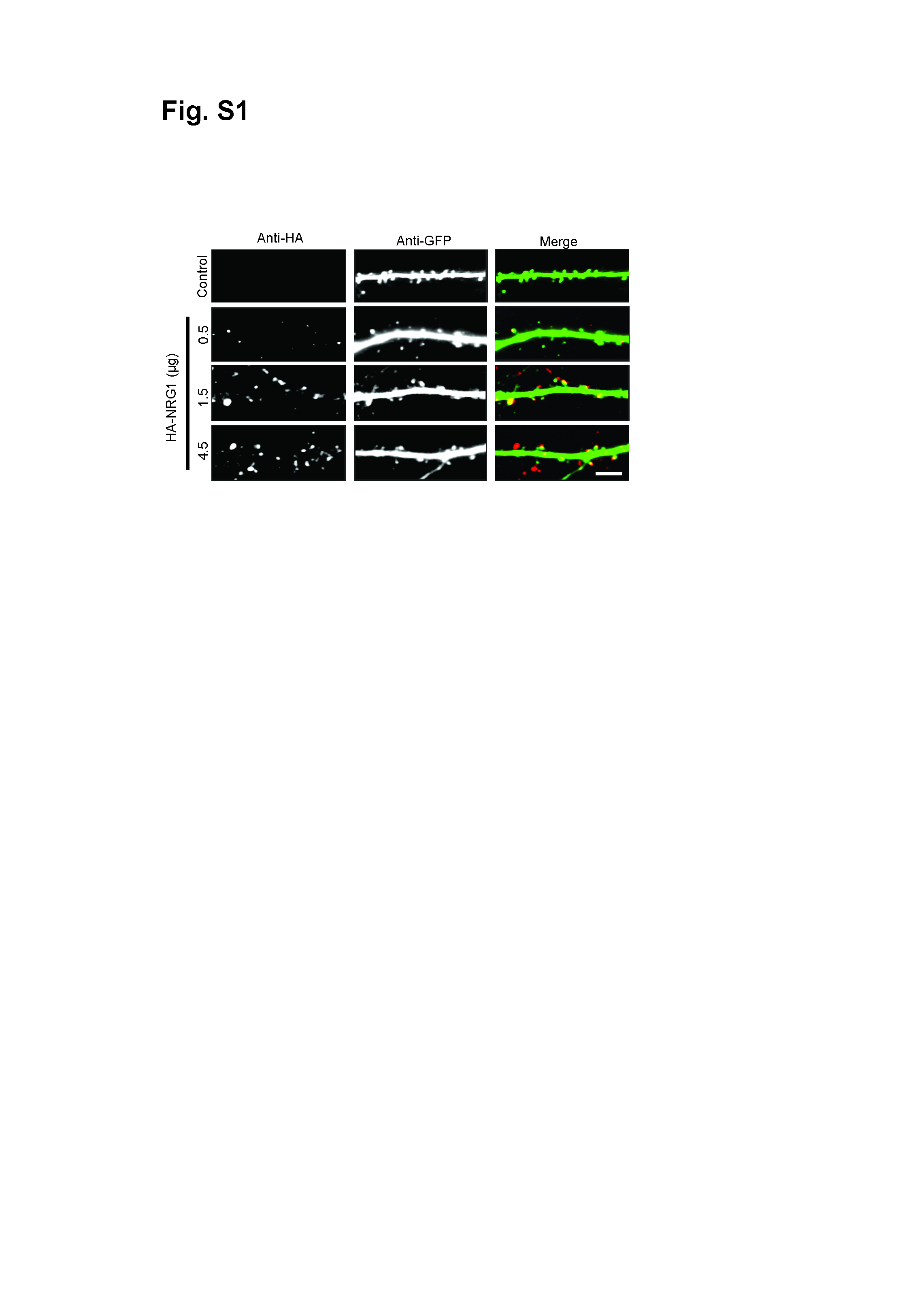

Supplement: Supplementary file 2 — Supplemental Figure 1 [file 41419_2021_3687_MOESM2_ESM.tif]

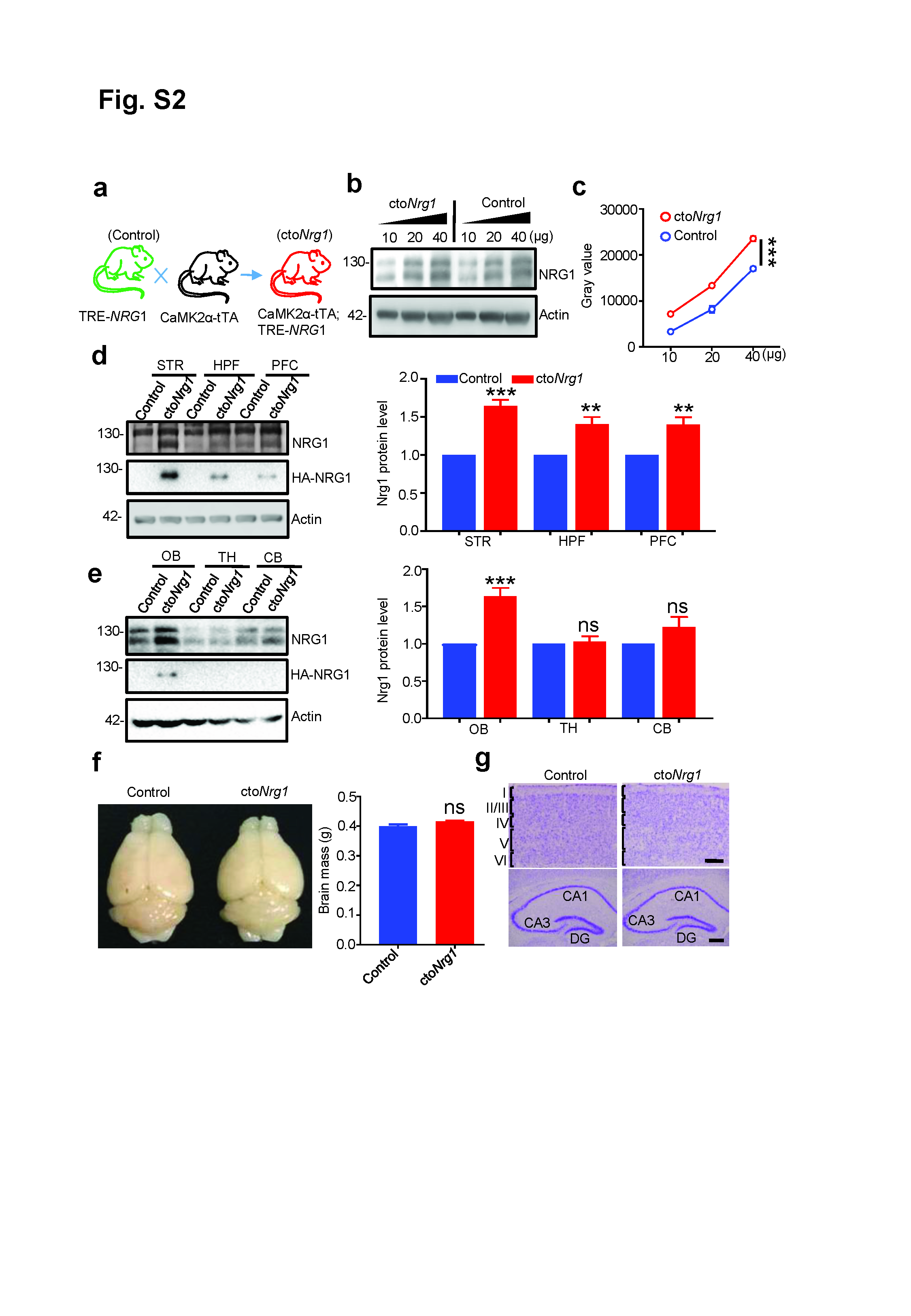

Supplement: Supplementary file 3 — Supplemental Figure 2 [file 41419_2021_3687_MOESM3_ESM.tif]

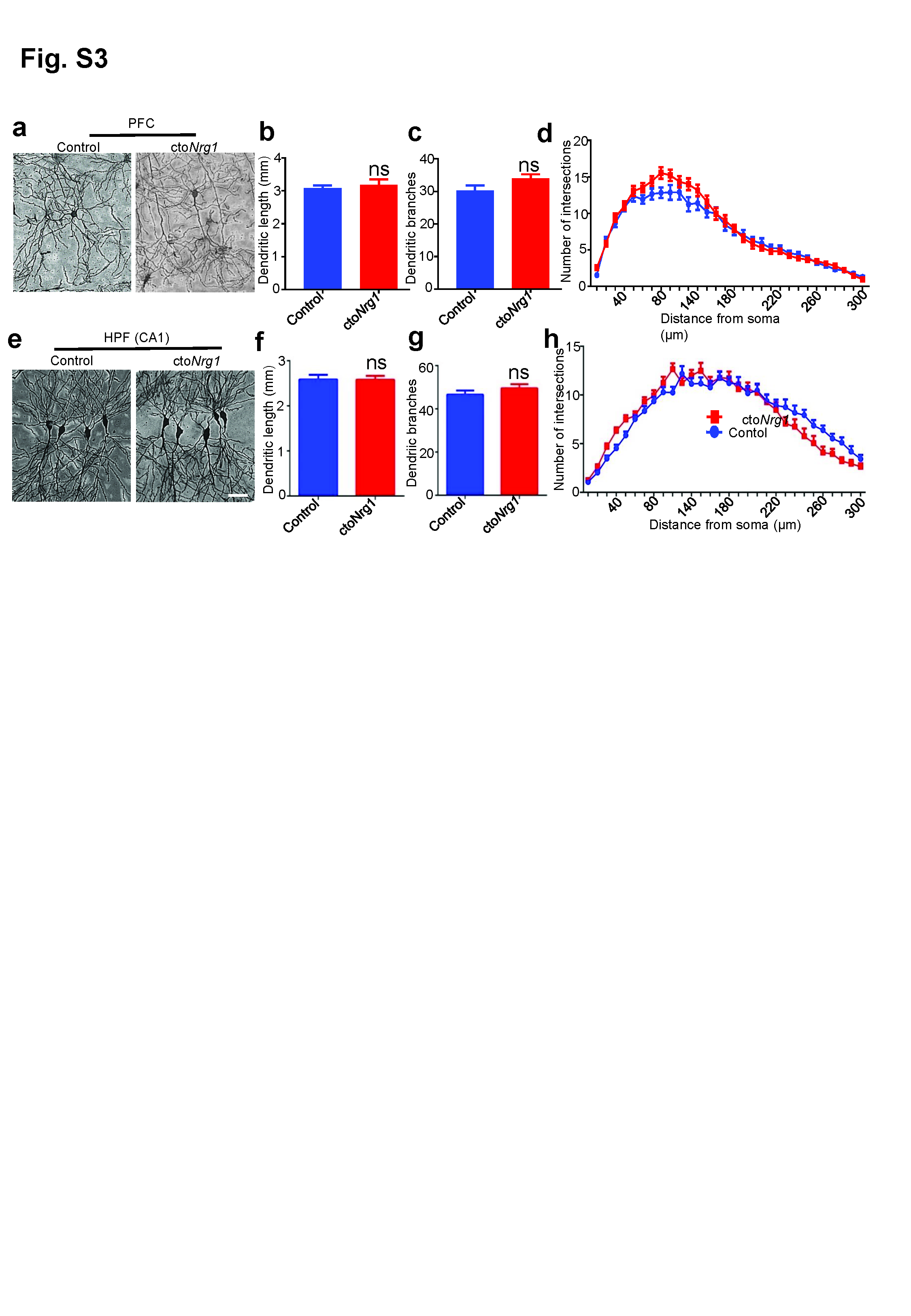

Supplement: Supplementary file 4 — Supplemental Figure 3 [file 41419_2021_3687_MOESM4_ESM.tif]

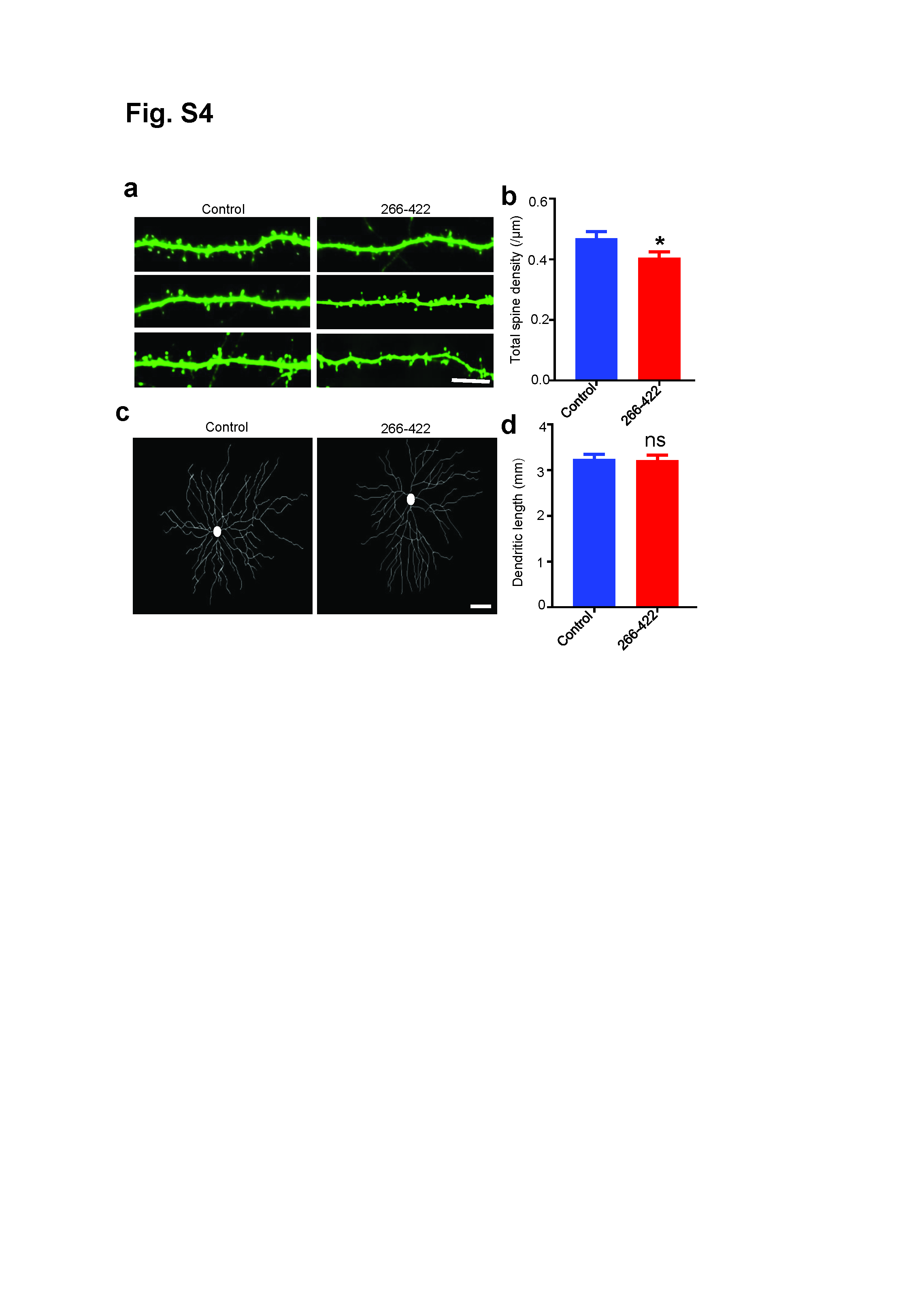

Supplement: Supplementary file 5 — Supplemental Figure 4 [file 41419_2021_3687_MOESM5_ESM.tif]
